# Nanoformulation Composed of Ellagic Acid and Functionalized Zinc Oxide Nanoparticles Inactivates DNA and RNA Viruses

**DOI:** 10.3390/pharmaceutics13122174

**Published:** 2021-12-16

**Authors:** Khaled AbouAitah, Abdou K. Allayh, Jacek Wojnarowicz, Yasser M. Shaker, Anna Swiderska-Sroda, Witold Lojkowski

**Affiliations:** 1 Laboratory of Nanostructures and Nanomedicine, Institute of High Pressure Physics, Polish Academy of Sciences, Sokolowska St. 29/37, 01-142 Warsaw, Poland; jacek.wojnarowicz@tlen.pl (J.W.); a.swiderska-sroda@labnano.pl (A.S.-S.); 2 Medicinal and Aromatic Plants Research Department, Pharmaceutical and Drug Industries Research Institute, National Research Centre (NRC), 33 El–Behouth St., Dokki, Giza 12622, Egypt; 3 Environmental Virology Laboratory, Water Pollution Research Department, Environment and Climate Change Institute, National Research Centre (NRC), 33 El–Behouth St., Dokki, Giza 12622, Egypt; drallayeh@yahoo.com; 4 Chemistry of Natural and Microbial Products Department, Pharmaceutical and Drug Industries Institute, National Research Centre (NRC), 33 El–Behouth St., Dokki, Giza 12622, Egypt; yabdelrahman11@yahoo.com

**Keywords:** nanoformulation/delivery system, antiviral, H1N1/human coronavirus, zinc oxide nanoparticles, natural agents, ellagic acid

## Abstract

The COVID-19 pandemic has strongly impacted daily life across the globe and caused millions of infections and deaths. No drug therapy has yet been approved for the clinic. In the current study, we provide a novel nanoformulation against DNA and RNA viruses that also has a potential for implementation against COVID-19. The inorganic–organic hybrid nanoformulation is composed of zinc oxide nanoparticles (ZnO NPs) functionalized with triptycene organic molecules (TRP) via EDC/NHS coupling chemistry and impregnated with a natural agent, ellagic acid (ELG), via non-covalent interactions. The physicochemical properties of prepared materials were identified with several techniques. The hybrid nanoformulation contained 9.5 wt.% TRP and was loaded with up to 33.3 wt.% ELG. ELG alone exhibited higher cytotoxicity than both the ZnO NPs and nanoformulation against host cells. The nanoformulation efficiently inhibited viruses, compared to ZnO NPs or ELG alone. For H1N1 and HCoV-229E (RNA viruses), the nanoformulation had a therapeutic index of 77.3 and 75.7, respectively. For HSV-2 and Ad-7 (DNA viruses), the nanoformulation had a therapeutic index of 57.5 and 51.7, respectively. In addition, the nanoformulation showed direct inactivation of HCoV-229E via a virucidal mechanism. The inhibition by this mechanism was > 60%. Thus, the nanoformulation is a potentially safe and low-cost hybrid agent that can be explored as a new alternative therapeutic strategy for COVID-19.

## 1. Introduction

The emergence of novel pathogenic viruses, microbes, and pandemic outbreaks is a challenge for humans. The novel coronavirus disease 2019 (COVID-19) pandemic is a current example showing the need for broad collaborative efforts in all areas of science and engineering to provide a better understanding and solutions [1]. Severe acute respiratory syndrome coronavirus 2 (SARS-CoV-2), causative agent of COVID-19, has quickly spread worldwide, since it was first detected in 2019 in China [2]. It has caused millions of infections and thousands of deaths, with a continual increase, creating an urgent demand to find antiviral drugs and vaccines. No specific drug therapy is yet available, but a rapid strategy is to evaluate antivirals exhibiting broad spectrum effects against other viruses [3]. As far as vaccines are concerned, some have been used during this outbreak that were made via different strategies. With an expectation of different strains of coronavirus, the need for new vaccines may be raised from time to time. As such, the current pandemic is a reminder of the urgent requirement for efficient antivirals, developed with an innovative strategy of detection, fluid filtration, surface disinfection, vaccines, and therapy [1].

Drug delivery systems (DDSs), employing nanoformulations, have been the main platform of nanomedicine over the last few decades, witnessing tremendous advancements in therapy against various infectious by viruses (i.e., HIV, influenza, coronaviruses) and bacteria [4,5,6,7]. The main concept behind using nano–antivirals is to allow nanoparticle (NP)–virus interactions, based on the small size of the virus, offering a realistic solution for treating them. As the nanomaterial/nanocarrier is the key factor in fabricating nanoformulations, many nanomaterials have been used, including carbon-based antiviral nanomaterials [8], mesoporous silica nanoparticles (NPs) [9], zinc oxide NPs [10], metal-organic frameworks [11], and silver NPs [12].

Of interest in the present study were ZnO NPs, due to their unique antimicrobial potential (with a different mode of action) and feasibility in tailoring any nanoformulations. In a recent study, we developed a hybrid nanoformulation, consisting of ZnO NPs impregnated with the natural agent of protocatechuic acid, which acts efficiently against the bacteria *Staphylococcus aureus* [13]. These promising results drove our team to continue using ZnO NPs to construct nanoformulations to control viral infections. Thus, we aimed to develop a novel hybrid (inorganic/organic) nanoformulation, composed of ZnO NPs functionalized with triptycene (TRP) and impregnated with the ellagic acid natural agent (ELG).

The TRP family is a unique class of three-dimensional aromatic structures, containing three separate arene (benzene blades) units, allowing π–π stacking or supramolecular interlocking via a strong π–π interaction [14]. TRP is a rigid and highly symmetric molecule with D_3h_ symmetry. These promising structural advantages have allowed the use of modern synthetic organic chemistry for the creation of new structures, such as new DDSs [15], and broad applications including material chemistry, molecular machines, supramolecular chemistry, crystal engineering, and nano-science [16,17,18,19]. These advantages inspired us to use the ZnO NPs-functionalized with TRP (nano inorganic–organic hybrids) as the core of the intended nanoformulation.

The natural prodrug ELG (2,3,7,8-tetrahydroxy-chromeno[5,4,3-cde]chromene-5,10-dione), a safe and powerful polyphenol, is present in several types of fruit (e.g., grapes, pomegranates, strawberries, and walnuts) [20,21] and medicinal plants [22]. It exhibits multiple pharmacological actions, including anticancer, antiviral, anti-inflammatory, and antimicrobial effects [21,23,24]. Elg could serve as a novel natural agent with efficient antiviral effects against several viruses, such as human rhinoviruses (e.g., HRV2, -3, and -4) [25], HIV-1 [26], herpes simplex virus (HSV) type 1 [27], Ebola [28], influenza [29], adenoviruses [30], and Zika virus [31]. Another recent strategy using Elg in antiviral research is its combination with clinically proven antiviral drugs to produce synergistic effects, such as anti-influenza effects with oseltamivir [32].

**Scheme 1:**
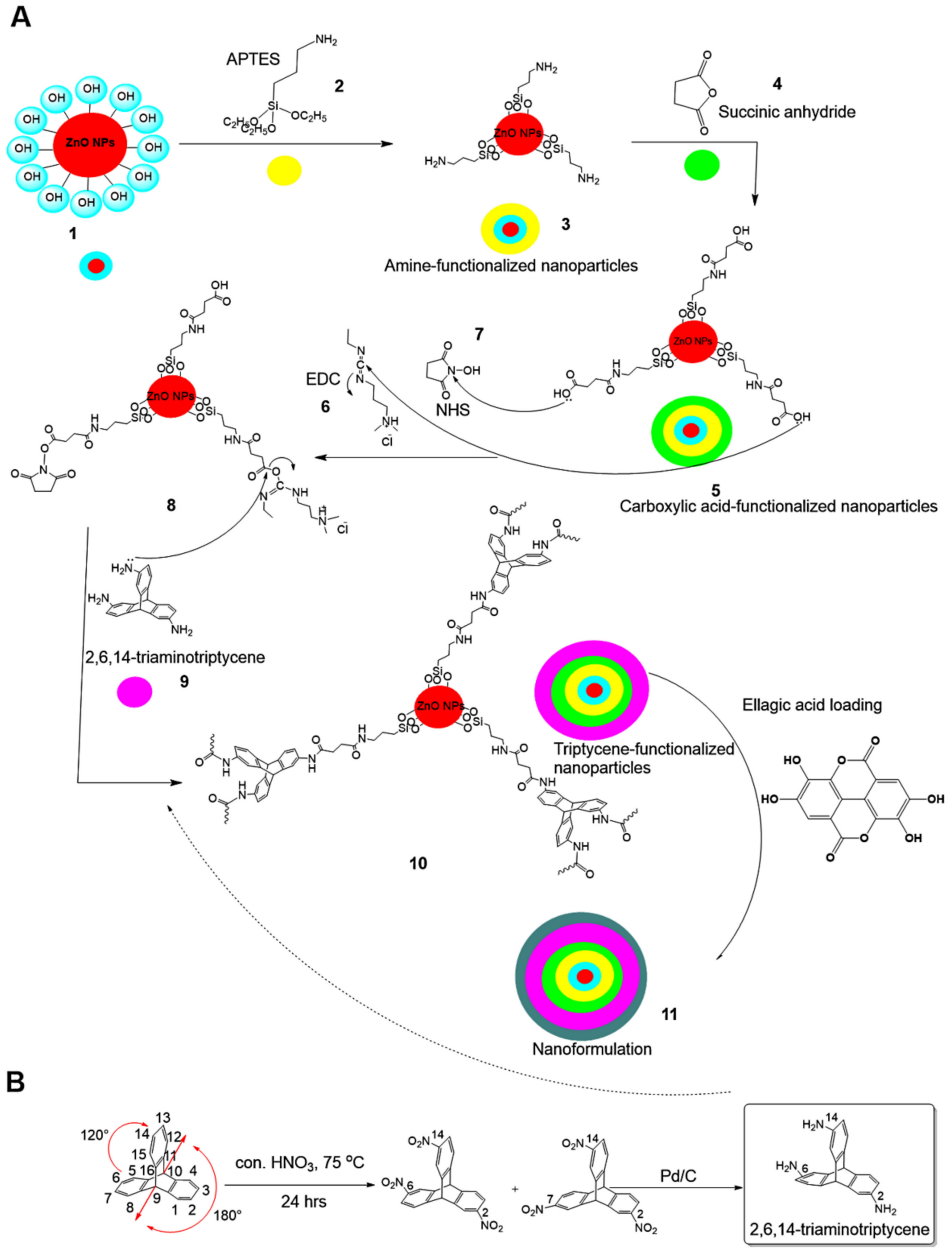
Preparation steps for the nanoformulation. (**A**) Synthesis of the nanoformulation from ZnO NPs (1) reacted with APTES silane molecules (2) to obtain amine-functionalized nanoparticles (3). The functionalized nanoparticles are further reacted with succinic anhydride (4), resulting in carboxylic acid–functionalized nanoparticles (5). The later nanoparticles are activated through 1-(3-dimethylaminopropyl)-3-ethylcarbodiimide hydrochloride (EDC, (6))/N-hydroxysuccinimide (NHS, (7)), leading to the coupling chemistry, shown in step (8). Triptycene (9) is then further reacted to obtain the hybrid inorganic/organic structure nanoparticles (10). Finally, the hybrid is used to load/impregnate the ellagic acid natural agent prodrug to reach the nanoformulation (11). (**B**) The full steps were followed to obtain triaminotriptycene (TRP).

Here, we report, for the first time, a smart nanoformulation design, characterized by an inorganic–organic hybrid, in which ZnO NPs (inorganic material) are coated with TRP (organic 3D material) and employed as the core. Subsequently, the core was impregnated with ELG. The constructed nanoformulation (Scheme 1) was tested for its specific antiviral effects to combat DNA viruses and RNA viruses. We employed several viral alternatives (human adenovirus (Ad-7) and herpes virus type 2 (HSV-2), human influenza (H1N1), and human coronavirus (HCoV-229E) as models to investigate the antiviral activity against COVID-19 in this study, for laboratory safety reasons. These alternatives, like COVID-19, cause respiratory disorders, with one of these alternatives, human coronavirus 229E, belong to the same family as SARS-CoV-2. In general, these viruses are the most common cause of respiratory illness in humans, with a considerable influence on morbidity and death across the world. The reason for testing different viruses is the unique structure of both classes of virus and expected various interactions with the nanoformulation. Overall, the nanoformulation showed promise as an antiviral agent against H1N1 and HCoV-229E, reflecting the possible implementation in designing nanomedicines against COVID-19.

## 2. Materials and Methods

### 2.1. Triptycene Functionalization

The ZnO NPs used in the present study were prepared according to the microwave-assisted solvothermal approach, described previously [33,34]. The TRP (2,6,14-Triaminotriptycene) was prepared starting from triptycene. Firstly, nitration of triptycene using conc. nitric acid was performed to obtain 2,6,14-trinitrootriptycene and 2,7,14-trinitrotriptycene constitutional isomers that successfully separated by column chromatography. Next, reduction 2,6,14-trinitrootriptycene was achieved using Pd/C in ethanol under refluxing, in order to obtain 2,6,14-triaminotriptycene (9), as shown in Scheme 1. The ZnO NPs were functionalized with TRP in a multi-step process:

1. In the first step, ZnO NPs were modified with amino groups (–NH_2_) by adding 1.250 g ZnO NPs to 150 mL anhydrous toluene (POCH, Gliwice, Poland) and reacting it with 1.5 mL 3-aminopropyltriethoxysilane (APTES, Sigma-Aldrich, St. Louis, MO, USA) for 24 h under medium stirring speed (250 rpm) at room temperature (RT). The surface-modified NPs were collected using a Sigma 3-30KS cooling centrifuge (Sigma Laborzentrifugen GmbH, Osterode am Harz, Germany) and washed with water (18.2 MW, Milli-Q^®^ system, Millipore, Darmstadt, Germany) to remove unreacted APTES. The collected surface-modified NPs were finally oven-dried at 50 °C to complete dryness, and the product was designated as ZnO NPs–NH_2_.
2. In the second step, we functionalized the ZnO NPs–NH_2_ with carboxylic groups (–COOH) through a modified synthesis protocol, according to Hakeem et al. [35]. ZnO NPs–NH_2_ were suspended in acetone (50 mL, Fisher Scientific, Loughborough, UK) under water-bath sonication (5 min, Elma GmbH, Singen, Germany) and stirred at RT for 4 h (DAIHAN Scientific, Seoul, Korea). Next, succinic acid anhydride (0.7 M, 40 mL, Acros Organics, Geel, Belgium), dissolved in acetone, was added dropwise under stirring (350 rpm) and allowed to react for 24 h at RT. Subsequently, the functionalized particles were collected by centrifugation and washed with both deionized water and methanol (Fisher Scientific, Loughborough, UK). Finally, the obtained particles were oven-dried for 12 h at 60 °C and designated as ZnO NPs–NH_2_–COOH.
3. In the final step, TRP-functionalized NPs were obtained in the following sequence: (1) ZnO NPs–NH_2_–COOH were activated through 1-(3-dimethylaminopropyl)-3-ethylcarbodiimide hydrochloride (EDC) and N-hydroxysuccinimide (NHS) coupling in 0.5% acetone (25 mL EDC and NHS, Acros Organics, Geel, Belgium) under stirring (400 rpm) for 6 h at 40 °C, resulting in solution A. (2) In separate screw cap bottles, TRP (300 mg) was activated in acetone containing EDC/NHS (0.5%, 50 mL), under stirring (400 rpm) for 6 h at 40 °C, resulting in solution B. (3) Solution B (25 mL) was slowly dropped into solution A and stirred (250 rpm) at RT for 24 h. (4) We separated the obtained NPs by centrifugation and washed them with deionized water and acetone to remove unreacted TRP, before drying at 60 °C. The obtained material was designated ZnO NPs–NH_2_–COOH–TRP.

### 2.2. Nanoformulation Preparation

To prepare the hybrid nanoformulation, we used a 1:1.5 drug to nanocarrier ratio. In a typical preparation, we dissolved 100 mg ELG (Sigma-Aldrich, St. Louis, MO, USA) in 15 mL of ethanol, followed by the addition of ZnO NPs–NH_2_–COOH–TRP (150 mg), and stirred (170 rpm) at RT for approximately 24 h. The solvent was evaporated at 50 °C in a Rotavapor (Büchi, Flawil, Switzerland) until dryness. We resuspended the obtained powder in 25 mL ultrapure water and evaporated it again. To ensure complete dryness, the nanoformulation powder was finally oven-dried for 12 h at 60 °C, yielding the final nanoformulation powder, designated ZnO NPs–NH_2_–COOH–TRP–ELG.

### 2.3. Characterization Techniques

In our study, the obtained materials were characterized by utilizing several different analyses. For visualization and identification of the elemental content, NPs were observed by field emission scanning electron microscopy (FE–SEM, Ultra Plus, Zeiss, Jena, Germany), which was coupled with energy-dispersive X-ray spectroscopy. The materials were sputtered with gold for imaging. The change in particle morphology was observed using scanning transmission electron microscopy (STEM) on an FEI TECNAI G2 F20 S-TWIN (Thermo Fisher Scientific, Waltham, MA, USA). We measured the specific surface area employing the Brunauer, Emmett, and Teller (BET) method depending on the ISO 9277:2010 (Gemini 2360, Micromeritics, Norcross, GA, USA). Before the measurements, each nanomaterial was degassed at 150 °C and the nanoformulation at 50 °C under constant helium flow for 24 h on a FlowPrep 060 desorption station (Micromeritics). The functional groups on the NP surface and nanoformulation were identified by Fourier transform (FT) infrared (IR) spectroscopy coupled with attenuated total reflectance (ATR) using the Bruker Tensor 27 IR instrument and Bruker Platinum ATR-Einheit A 255 (Bruker Corporation, Billerica, MA, USA), respectively. We employed a coupled analysis for thermogravimetry (TG) and differential scanning calorimetry (DSC) to investigate the thermal stability and structure of all prepared materials through simultaneous thermal analysis (STA) on a 449 F1 Jupiter^®^ (NETZSCH-Feinmahltechnik GmbH, Selb, Germany). For this aim, the materials were heated, starting from RT and increasing 10 °C/min, to 800 °C in an artificial air and helium mixture within the furnace chamber. Before heating the furnace, the chamber was purged with the same gas mixture during 10 min. We determined the zeta potential of the suspended particles in deionized water, adjusted to pH 7.4, using a laser Doppler electrophoresis (LDE) analyzer (λ = 633 nm, Zetasizer Nano-ZS ZEN 3600, Malvern Instruments Ltd., Malvern, UK). To monitor the distribution of the particle size of materials suspended in deionized water, we performed particle tracking analysis (PTA) using the NS500 NanoSight instrument (λ = 405 nm, Malvern Panalytical Ltd., Malvern, UK).

### 2.4. Antiviral Evaluations

#### 2.4.1. Viruses and Cell Lines

We used several viruses and cell lines, purchased from American Type Culture Collection (ATCC, Manassas, VA, USA) and kindly provided by Nawah-Scientific Co., Cairo, Egypt. The host cell lines were head and neck cancer cells (Hep-2) for propagation of human adenovirus type 7 (Ad-7), green monkey kidney (Vero) cells for propagation of herpes simplex virus type 2 (HSV-2), and clone of Vero (Vero-E6) cells for propagation of human influenza (H1N1) and human coronavirus 229E (HCoV-229E). The cells were grown in DMEM medium-high glucose (Grand Island, NY, USA) containing 10% fetal bovine serum (Grand Island, NY, USA), 0.1% antibiotic/antimycotic solution (Gibco BRL, Grand Island, NY, USA), and trypsin-EDTA (Grand Island, NY, USA).

#### 2.4.2. Cytotoxicity Assessment

To assess the cytotoxicity of all materials, we performed the sulforhodamine B (SRB) colorimetric assay as described previously [36]. We seeded Hep–2, Vero, and Vero–E6 cells in 96-well culture plates at a density of 2 × 10^4^ cells/well and allowed them to grow for 24 h. We added serially diluted samples in culture medium to the wells and incubated them for another 48 h. Next, we removed the medium, washed the cells with phosphate buffered saline (PBS), and added 0.01 mL of cold acetone (70%, *v*/*v*) to each well. The plates were left at −20 °C for 30 min. We removed the acetone and oven-dried the 96-well plates for 30 min at 60 °C. In the next step, we added 0.01 mL of SRB (0.4% (*w*/*v*)) in 1% acetic acid (*v*/*v*) to each well and left the plates at RT for 30 min. We washed the plates with 1% acetic acid (*v*/*v*) five times to remove unbound SRB and allowed them to dry. Subsequently, we solubilized the fixed SRB by adding 100 µL of unbuffered Tris base solution (10 mM) to the wells at RT for 30 min. Finally, we used a microplate reader (BMG LabTech GmbH FLUOstar Omega, Ortenberg, Germany) to determine the optical density (OD) at 540 nm. The reference absorbance was obtained at 620 nm. The 50% cytotoxic concentration (CC50) was calculated through the GraphPad PRISM (Version 5, GraphPad Software, San Diego, CA, USA).

### 2.5. Antiviral Assessment

#### 2.5.1. Cytopathic Assay

The cytopathic inhibition effect (CPE) was used to evaluate the antiviral effect, according to Song et al. [37]. Cells were seeded at a density of 2 × 10^4^ cells/well in 96-well culture plates. They were allowed to grow for 24 h before infection. Next, the culture medium was removed, and the cells were washed with PBS. We added 0.1 mL of each diluted virus suspension to the mammalian cell line. This suspension contained the 50% cell culture infective dose of virus stock, in order to produce the desired CPEs post-infection. For compound treatments, we added 0.01 mL of medium containing the desired concentration of material to the cells. Each test sample’s antiviral activity was determined using the 10-fold diluted concentration range (0.1–100 µg/mL). From 3 to 4 days, according to virus type, culture plates were incubated in a 5% CO_2_ atmosphere at 37 °C. We removed the medium and washed the cells with PBS before adding 0.01 mL of 70% (*v*/*v*) cold acetone to each well. The wells were kept at −20 °C for 30 min. Thereafter, we removed the acetone and oven-dried the 96-well plates at 60 °C for 30 min. In the next step, we added 0.01 mL of SRB (0.4% (*w*/*v*)) in 1% acetic acid (*v*/*v*) to each well and left the plates at RT for 30 min. We washed the plates with 1% acetic acid (*v*/*v*) five times to remove unbound SRB and allowed them to dry. Subsequently, we solubilized the fixed SRB by adding 100 µL of unbuffered Tris base solution (10 mM) to the wells at RT for 30 min. Finally, we used a microplate reader (BMG LabTech GmbH FLUOstar Omega, Ortenberg, Germany) to determine the OD at 540 nm. The reference absorbance was obtained at 620 nm, and the 50% inhibitory concentrations (IC50) was calculated through the GraphPad PRISM (Version 8.0.1, GraphPad Software, San Diego, CA, USA).

#### 2.5.2. Virucidal Mechanism

We used the plaque reduction assay to explore the viricidal potential as described previously [38]. We seeded Vero E6 cells at a density 10^5^ cells/mL in a 6-well plate and allowed them to grow for 24 h in a 5% CO_2_ atmosphere at 37 °C. Before the incubation of coronavirus with cells, the HCoV-229E virus was diluted to 10^3^ PFU and a 100 µL aliquot added to 100 µL of the tested materials (at their IC50s). We incubated the mixture for 1 h and then added to the Vero E6 cells in the 6-well plate. After 1 h (contact time), we removed the supernatant and added 3 mL of DMEM medium (supplemented with 2% agarose). We left the plates to solidify at 37 °C. Viral plaques were fixed and stained using the following steps. We added 10% formalin to each well and removed the overlayer after 1 h. We stained the formalin-fixed cells with crystal violet (0.1% in distilled water). In each plate, we used the untreated virus as a control. Finally, we counted the formed plaques and calculated the reduction in HCoV–229 as the % inhibition using the formula: viral count (untreated)—viral count (treated)/viral count (untreated) × 100.

### 2.6. Statistical Analysis

Two-way analysis of variance (ANOVA) was employed for cytotoxicity and antiviral evaluations. Calculations were carried out using GraphPad PRISM (Version 8.0.1, GraphPad Software, San Diego, CA, USA).

## 3. Results and Discussion

### 3.1. Hybrid Nanoformulation Preparation

The nanoformulation preparation was achieved in the steps as shown in Scheme 1. To carry out the surface modification of ZnO NPs **1** with amine functionalization, we used 3-aminopropyltriethoxysilane (APTES) **2** after toluene formed amine-functionalized NPs (ZnO NPs–NH_2_, **3**). Next, ZnO NPs–NH_2_ were reacted with succinic anhydride **4** for transformation to the carboxylic acid-functionalized NPs (ZnO NPs–NH_2_–COOH, **5**). ZnO NPs–NH_2_–COOH was treated via coupling chemistry with EDC **6** and NHS **7** as coupling agents to successfully obtain the *O*-acylisourea active ester **8** for further modification. This *O*-acylisourea active ester is easily displaced by nucleophilic attack by amino groups of primary amines forming amide bonds via coupling reaction. ion. 2,6,14-Triaminotriptycene (Trp) **9** was chosen for the coupling reaction with ZnO NPs–NH_2_–COOH via the *O*-acylisourea active ester intermediate in order to form triptycene based polyamide networks tagged ZnO NPs for further functionalized modification of the nanoparticles through three-dimensional structure **10**. The full preparation of TRP molecules is presented in Scheme 1B. The coupling reaction between TRP and the active ester formed TRP-functionalized NPs (ZnO NPs–NH_2_–COOH–TRP, 10), resulting in three–dimensional hybrid inorganic–organic NPs that could be used for loading ELG prodrug molecules. Loading ELG **11** onto TRP-modified NPs resulted in the nanoformulation containing Elg agent (ZnO NPs–NH_2_–COOH–TRP–ELG, **12**).

**Figure 1:**
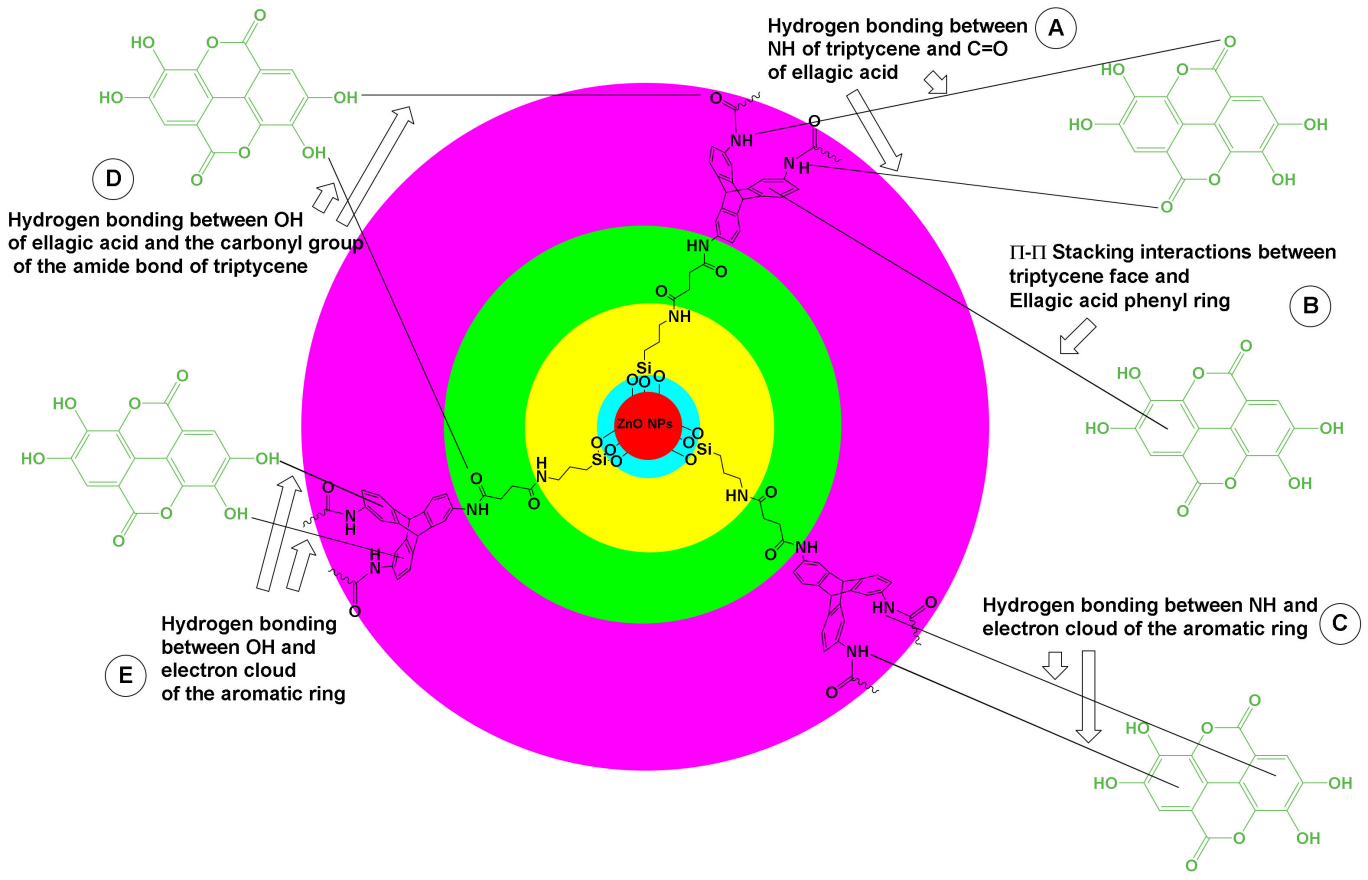
The possible link between the hybrid inorganic–organic nanoparticles and ELG molecules to form the nanoformulation. (**A**) Proposed hydrogen bonding between NH and C=O. (**B**) π–π stacking. (**C**) Hydrogen bonding between the NH and electron clouds. (**D**) Hydrogen bonding between OH and the carbonyl group. (**E**) Hydrogen bonding between OH and the electron clouds.

We propose that the interaction of ELG with TRP-functionalized NPs occurred through various intermolecular noncovalent interactions (Figure 1): (1) hydrogen bonding between the N–H groups of TRP and carbonyl units of ELG; (2) π–π stacking between benzene blades of TRP and the benzene rings of ELG; (3) hydrogen bonding between the N–H groups of TRP and electron clouds of the benzene rings of ELG; (4) hydrogen bonding between the phenolic hydroxyl groups of ELG and carbonyl group of the amide bond of TRP; and (5) hydrogen bonding between the phenolic hydroxyl groups of ELG and electron clouds of the benzene blades of TRP.

### 3.2. Microscopic Observations

**Figure 2:**
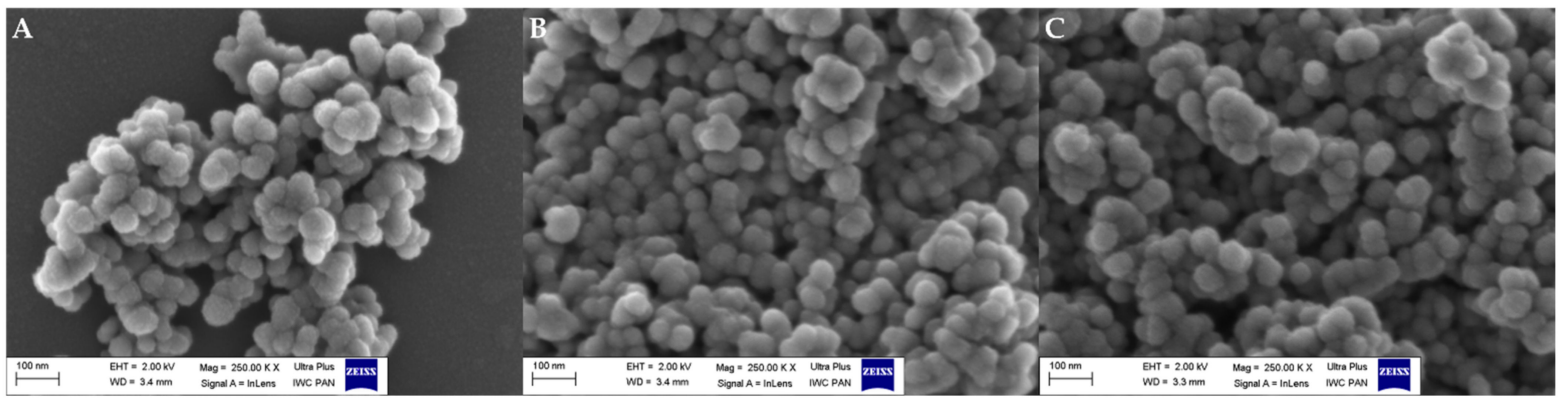
FE–SEM images of (**A**) ZnO NPs, (**B**) ZnO NPs–NH_2_–COOH–TRP, and (**C**) ZnO NPs–NH_2_–COOH–TRP–ELG.

**Figure 3:**
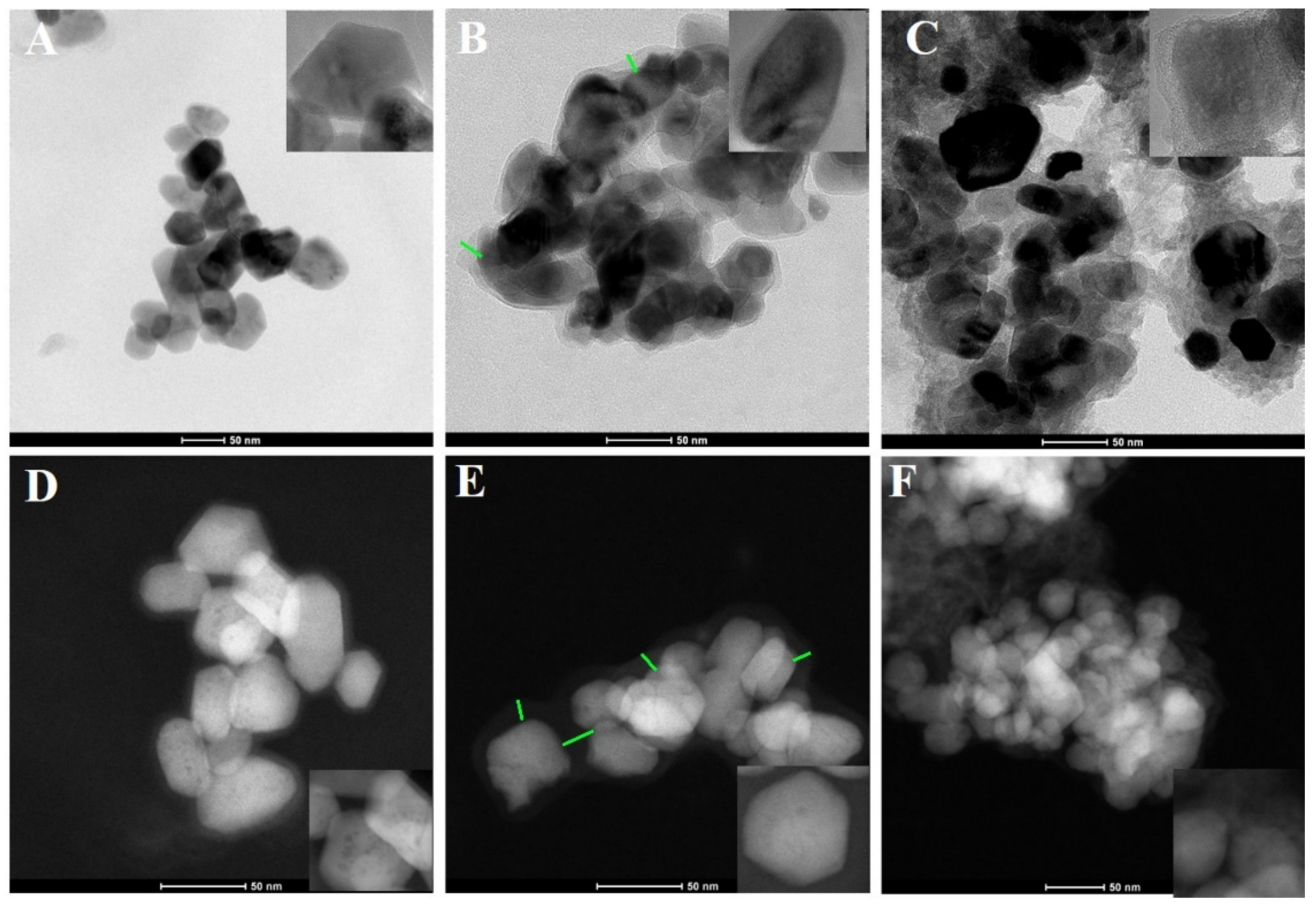
The morphological structure when using STEM as the material after surface functionalization and ELG loading. (**A**) The images were taken by TEM for pristine ZnO NPs, (**B**) ZnO NPs–NH_2_–COOH–TRP, and (**C**) ZnO NPs–NH_2_–COOH–TRP–ELG. (**D**) The images were taken by STEM for ZnO NPs, (**E**) ZnO NPs–NH_2_–COOH–TRP, and (**F**) ZnO NPs–NH_2_–COOH–TRP–ELG. The green lines show the coating shell layer, as indicated by TRP-functionalized nanoparticles.

In regard to observing changes at the morphological level, the main reason was to verify the presence of TRP on/in the ZnO NPs. The materials before and after the attachment of TRP and ELG loading were visualized by FE–SEM. The images showed no substantial differences in the particle morphology between ZnO NPs, ZnO NPs–NH_2_–COOH–TRP, and ZnO NPs–NH_2_–COOH–ELG (Figure 2). We sought to confirm this with another analysis, so we observed these materials by TEM and STEM. Interestingly, the coating/embedding of TRP onto NPs was observed. TEM images (Figure 3A–C) show a coating shell layer (green lines) covering the NPs (Figure 3B), compared to pristine ZnO NPs (Figure 3A), and the layer was present after ELG loading (Figure 3C). This condition was also confirmed by STEM analysis (Figure 3D–F). Overall, both TEM and STEM revealed the successful functionalization of ZnO NPs–NH_2_–COOH with TRP, as well as the possibility of obtaining an inorganic–organic hybrid nanostructure with porosity to assist in ELG impregnation for drug delivery applications.

### 3.3. Particle Size Measurements

**Figure 4:**
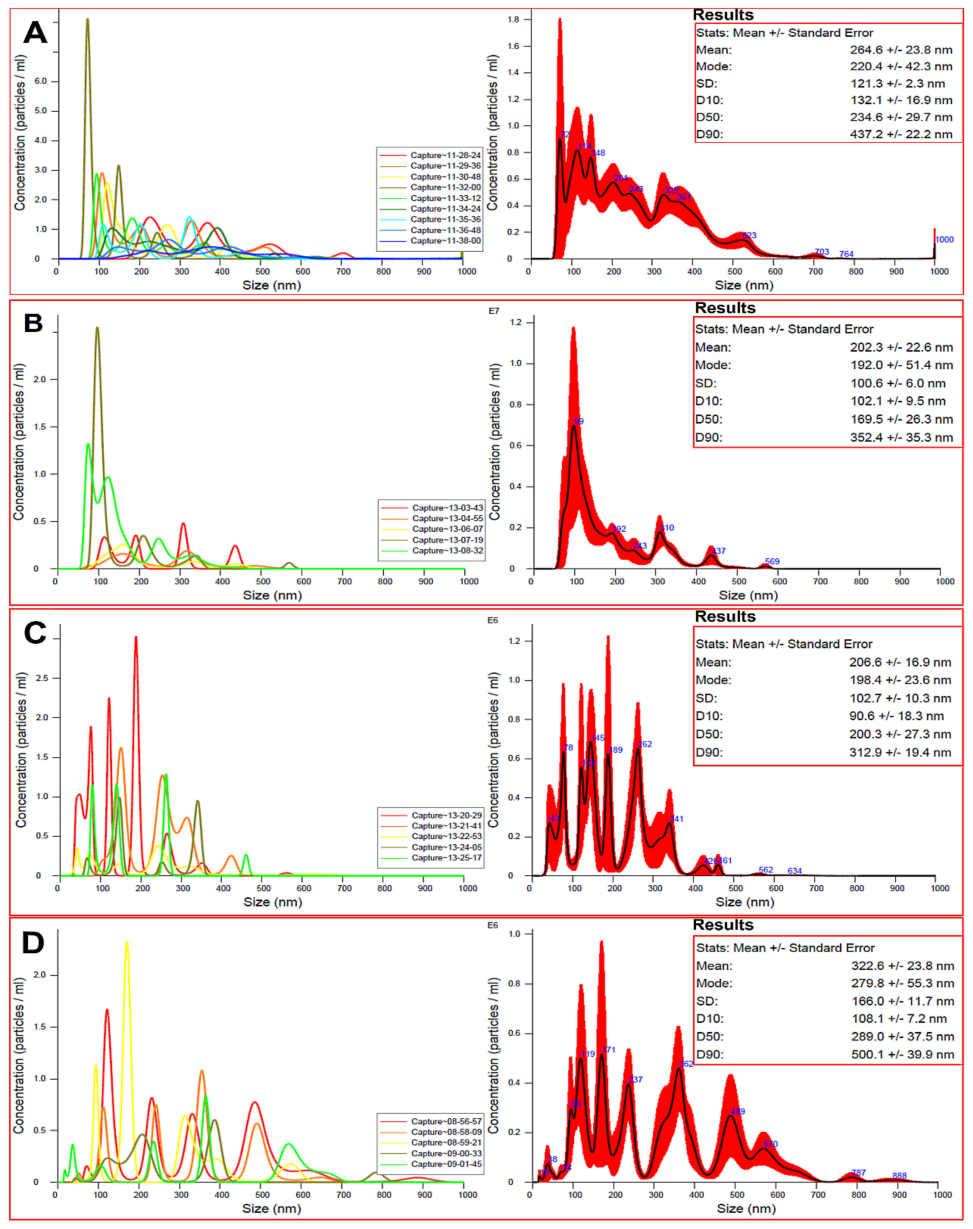
The particle size distribution determined by particle tracking analysis (PTA). (**A**) ZnO NPs; (**B**) ZnO NPs–NH_2_–COOH; (**C**) ZnO NPs–NH_2_–COOH–TRP; and (**D**) ZnO NPs–NH_2_–COOH–TRP–ELG.

The particle size of the nanocarrier material is important to consider when constructing any nanoformulations because it governs many functions, such as interactions with the biological entities, affecting their molecular, cellular, and organ levels. As our study concerns tiny biological entities, such as viruses, we measured the size distribution by PTA. In this context, PTA allows visualization of NPs, providing the size, count, and concentration measurements [39]. The PTA (Figure 4) found varied size distribution following the preparation steps, compared to the pristine ZnO NPs, with a mean size of 264.6 nm ± 23.8 (Figure 4A). For ZnO NPs–NH_2_–COOH, the mean size decreased to 202.3 nm ± 22.6 nm (Figure 4B). This observation may be related to improved dispersion stability of particles in aqueous solution, due to the surface functionalization with carboxylic acid groups [40]. As expected, the attachment of TRP in ZnO NPs–NH_2_–COOH–TRP led to an increase in the mean size, to 206.0 nm ± 16.9 nm, but still smaller than ZnO NPs (Figure 4C). The maximum size distribution was recorded for ELG loaded NPs in ZnO NPs–NH_2_–COOH–TRP–ELG, with a mean size of 322. nm ± 23.8 nm (Figure 4D). Thus, the particle size distribution is modulated by different parameters, such as surface functionalization and drug loading [9,41].

### 3.4. Specific Surface Area

We measured the specific surface area of materials before and after surface modification and ELG loading by BET analysis (Table 1). We found that the specific surface area was affected by several preparation steps. As expected, it decreased from 51.2 m^2^g^−1^ (ZnO NPs) to 21.6 m^2^g^−1^, 13.1 m^2^g^−1^, and 11.7 m^2^g^−1^ for ZnO NPs–NH_2_–COOH, ZnO NPs–NH_2_–COOH–TRP, and ZnO NPs–NH_2_–COOH–TRP–ELG, respectively.

### 3.5. Zeta Potential Measurements

**Figure 5:**
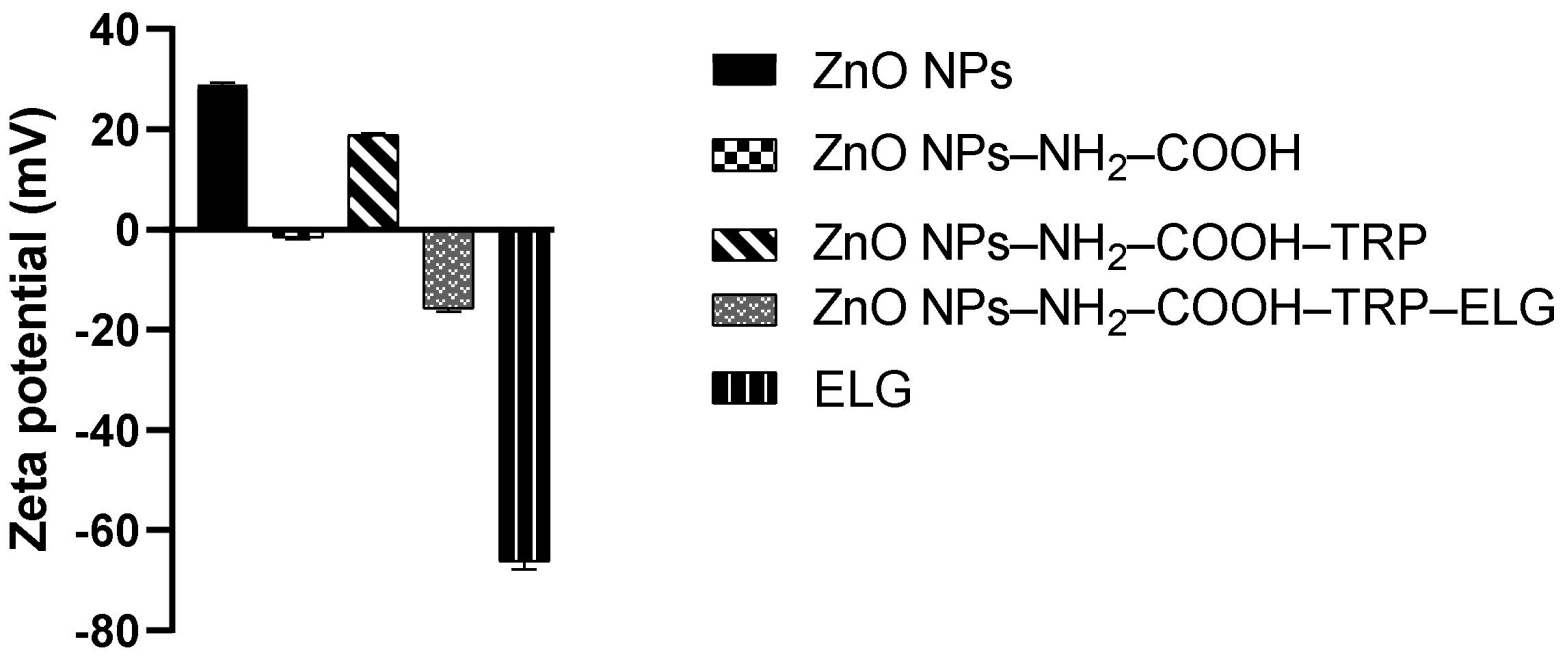
Zeta potential measurements of materials at different stages. The measurements were performed using the suspended solution of each material in deionized water, adjusted to pH 7.4.

The zeta potential changes were measured for NPs (at different stages) suspended in deionized water. Figure 5 shows that the surface modification by carboxylic groups (ZnO NPs–NH_2_–COOH) changed the surface from a positive zeta potential (28.9 mV ± 0.4 mV) in ZnO NPs to a negative zeta potential (−1.60 mV ± 0.24 mV). Further attaching the Trp molecules to the carboxylic acid-modified NPs changed the surface back to a positive zeta potential, as indicated by ZnO NPs–NH2–COOH–TRP (18.9 mV ± 0.32 mV). The results agree with our recent study on ZnO NPs functionalized with various silanes, in which the zeta potential of NPs differed as the type of functionality changed [13]. By loading ELG, the surface of NPs gained a negative zeta potential (−15.8 mV ± 0.5 mV) in ZnO NPs–NH2–COOH–TRP–ELG). This observation is expected, due to the negative zeta potential of Elg (−66.2 mV ± 1.5 mV), which is in line with what we observed when loading protocatechuic acid onto modified ZnO NPs in our previous study [13]. Taken together, the findings indicate that the surface functionality modulates the zeta potential of the surface of ZnO NPs, affecting various aspects, such as cytotoxicity [42] and antimicrobial action [43].

### 3.6. FTIR–ATR Analysis

**Figure 6:**
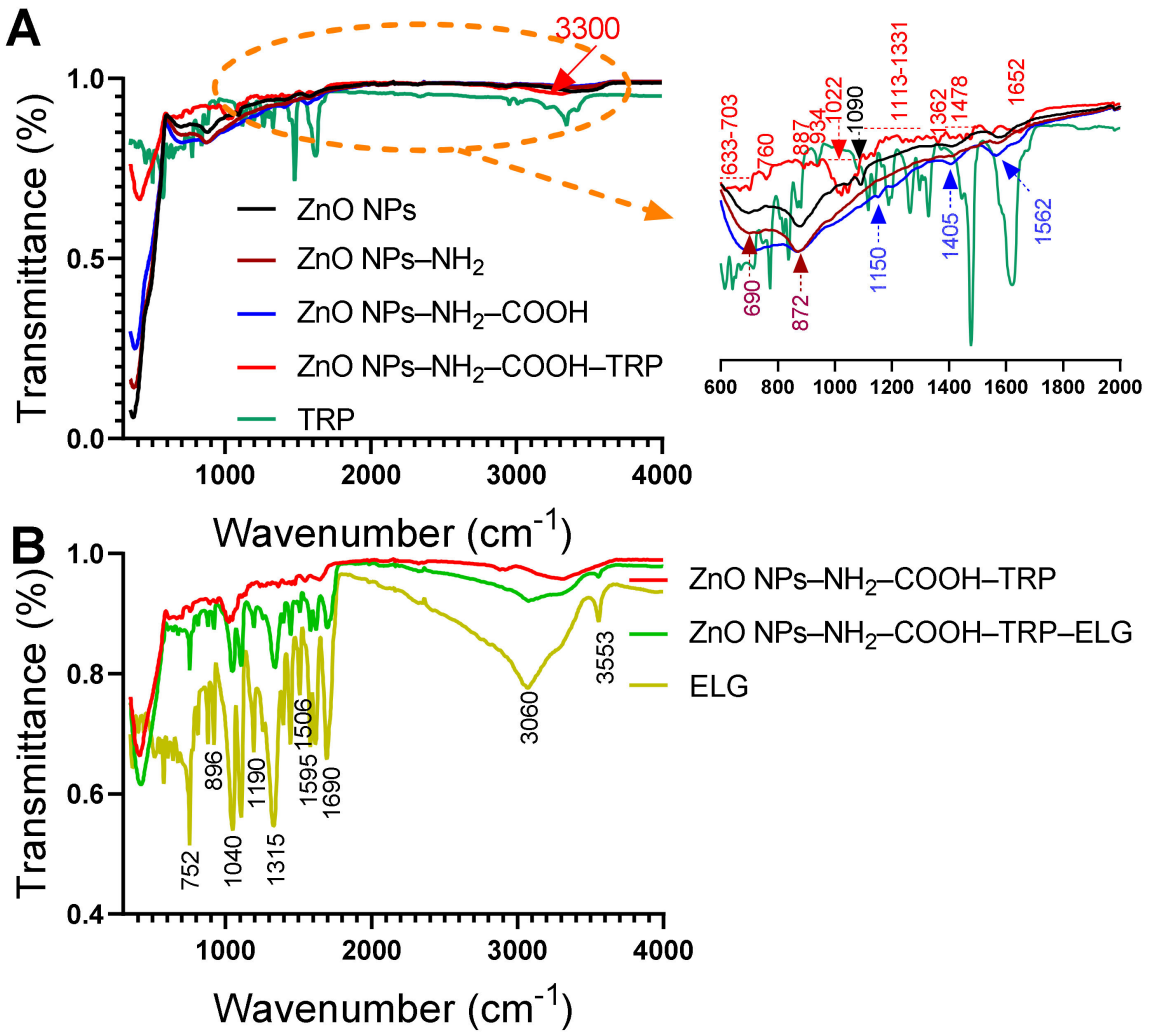
FT–IR analysis of materials. (**A**) FT–IR spectra show the materials before and after surface functionalization with amino groups, carboxylic acid, and TRP. (**B**) FT–IR spectra show the materials before and after ELG loading. The powder was analyzed for each material, with an ATR unit of the FT–IR device.

The FTIR–ATR spectra for ZnO NPs, before and after surface functionalization, are shown in Figure 6A. The most changes were detected in the range of 600–2000 cm^−1^. We used the similar parent ZnO NPs reported in our previous study, which are characterized by several distinguished bands (to not repeat, please refer to data in [13]). Compared to ZnO NPs, the amino-modified spectrum showed a relatively small increase in intensity at 690 cm^−1^ and 872 cm^−1^, whereas the peak at 1090 cm^−1^ disappeared. The band at 690 cm^−1^ may be attributed to Si–C streching vibration. The band at 872 can be assigned to Si–O–Si asymmetric stretching [44]. The appearance of these peaks confirms the attachment of amine groups from the APTES terminal silane to the surface of ZnO NPs [13,44,45]. Further modifying the amine-modified NPs with succinic acid anhydride, to obtain the carboxylic functional groups, resulted in new peaks. As seen in the FT–IR spectrum for ZnO NPs–NH_2_–COOH, peaks occurred at 1150, 1405, and 1562 cm^−1^, indicating the presence of carboxylic groups [46]. The peak at 1150 cm^−1^ can be related to C–O–C stretching, and the latter two peaks can be assigned to symmetric and antisymmetric COO^−^ stretching [47]. After attaching TRP, new bands indicating free TRP in the spectra range between 600 to 1700 cm^−1^ appeared. These peaks strongly indicate the attachment of TRP and suggest an interaction between TRP and carboxylic-modified NPs. The region between 1000 and 1150 cm^−1^ can be corresponded to the stretch vibration of C–N bond. The main peaks at 1022 cm^−1^ and 3300 cm^−1^, indicating the amide bond formation. Consequently, we obtained the inorganic–organic hybrid material with the porous structure on the surface, due to attachement of TRP. We appear to be the first to report this nanohybrid structure. FT–IR data in line with TEM and STEM observations confirm that TRP is present on the surface of carboxylic acid-modified ZnO NPs. Figure 6B shows the differences in the functional groups in TRP-modified NPs, the nanoformulation, and free ELG. Several peaks, especially at 752, 896, 1040, 1190, 1315, 1506, 1595, 1690, 3060, and 3553 cm^−1^, were detected in the nanoformulation that correspond to free ELG, confirming ELG loading into/on TRP-modified NPs. Among other peaks, the peak at 1315 cm^−1^ can be related to the carboxyl group (C=O) stretching present in ELG, and the peak at 1690 cm^−1^ can be assigned to C=C stretching [48,49]. In addition, two peaks centered at 3060 cm^−1^ and 3553 cm^−1^ may correspond to O–H stretching vibrations and the carboxyl group in ELG. These data agree with our recent results for protocatechuic acid loaded on the surface of functionalized ZnO NPs [13].

### 3.7. Thermal and DSC Analysis

**Figure 7:**
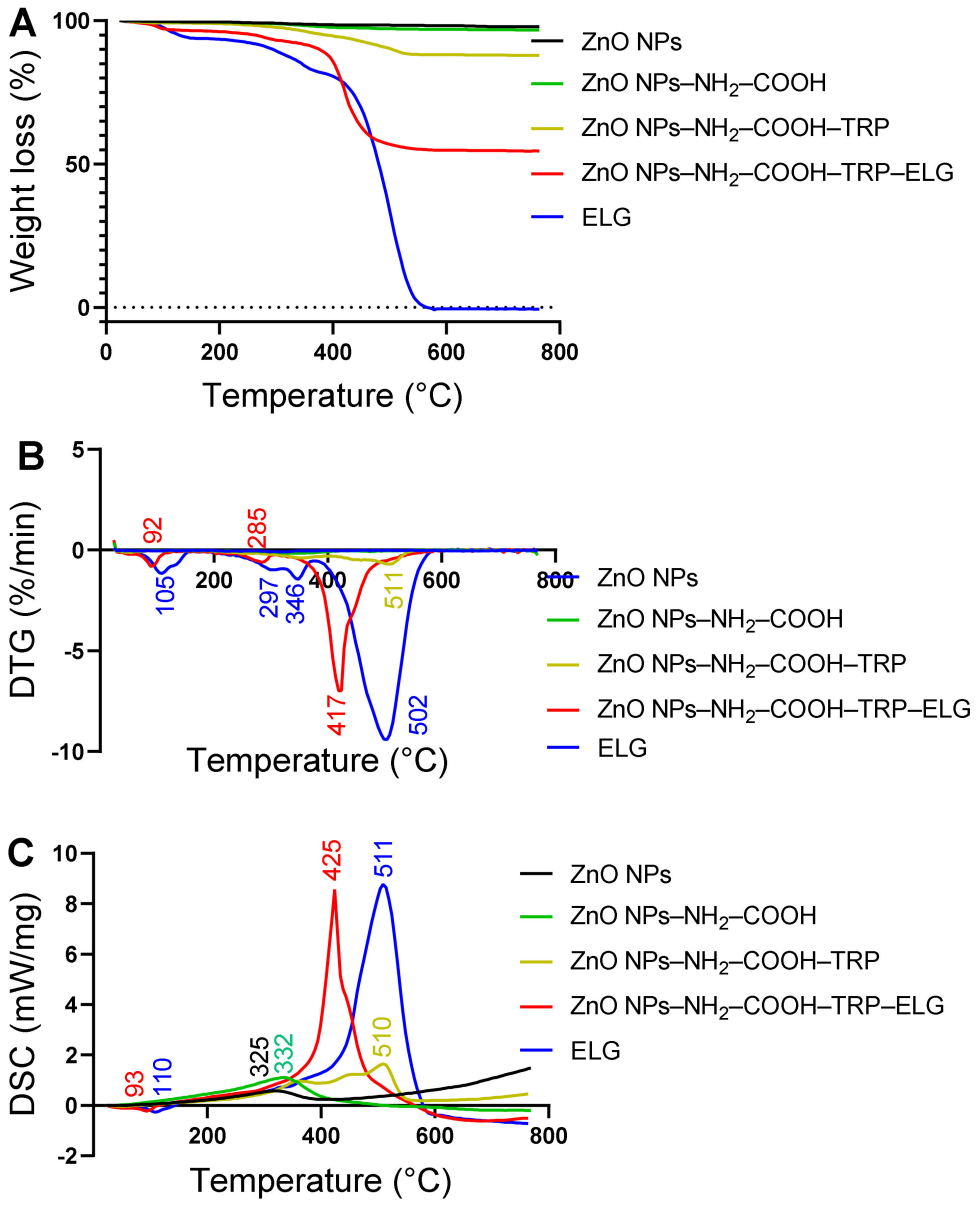
Thermal properties of materials at different stages, characterized using STA with DSC. (**A**) Weight loss measurements of all materials using STA. (**B**) DTG thermograms of all materials. (**C**) DSC analysis of all materials.

The thermal properties of materials at each preparation step are shown in Figure 7. As indicated by weight loss mass (Figure 7A), parent ZnO NPs and ZnO NPs–NH_2_–COOH (carboxylic acid-modified NPs) presented similar weight loss characteristics. As seen in Table 1, the mass loss did not change significantly between the two materials, with 2.2 and 3.3 wt.% for ZnO NPs and ZnO NPs–NH_2_–COOH, respectively. In contrast, ZnO NPs–NH_2_–COOH–TRP had significant mass loss (12.1 wt.%), with the main intensification of mass change kinetics between 320–550 °C and a peak centered at 511 °C, as indicated by the derivative thermogravimetric (DTG) curve (Figure 7B). The mass loss in this material is related to the decomposition of Trp molecules as organic matter. This mass loss shows the amount of TRP attached to NPs, which is likely as a surface coating. As expected, pure ELG decomposed (100 wt.%) in a manner characterized by three process steps (Figure 7A). As indicated by the DTG data (Figure 7B), the first decomposition was related to the removal of water, with a peak centered at ~105 °C; the second decomposition occurred at ~297 °C and 346 °C; the last main process at ~502 °C, relating to organic substance decomposition. The nanoformulation showed significant mass loss (~45.5 wt.%) and exhibited three phases of changing mass, as shown in the DTG pattern. The first phase of mass change, with a peak at ~92 °C, was associated with water evaporation from the nanoformulation, followed by small intensive decomposition at ~285 °C and, eventually the main decomposition at ~417 °C, which we attributed to the ELG decomposition. The total drug content of ELG in the nanoformulation was calculated to be > 33 wt.%, and the was entrapment efficiency ~83%, based on the weight loss principle (Table 1).

Figure 7C shows that an exothermic process upon heating the materials was related to the mass loss and DTG results (Figure 7A,B). Broad exothermic peaks were seen for ZnO NPs and ZnO NPs–NH_2_–COOH at ~325 °C and ~332 °C, respectively. After surface modification with TRP, a broad peak centered at ~510 °C in ZnO NPs–NH2–COOH–TRP indicated the attachment of TRP on the surface. For pure ELG, a small endothermic (down) peak appeared at ~110 °C, which is attributed to water evaporation; a second sharp intensive exothermic peak (up) was observed at ~511 °C, corresponding to the decomposition of ELG. For the nanoformulation, two peaks were detected: one endothermic at ~93 °C, due to water evaporation, and the second sharper intense exothermic at ~425 °C, due to ELG decomposition in the nanoformulation. Notably, the DCS curves correlate with DTG data, indicating the kinetics of mass change.

### 3.8. Cytotoxicity Evaluations

**Figure 8:**
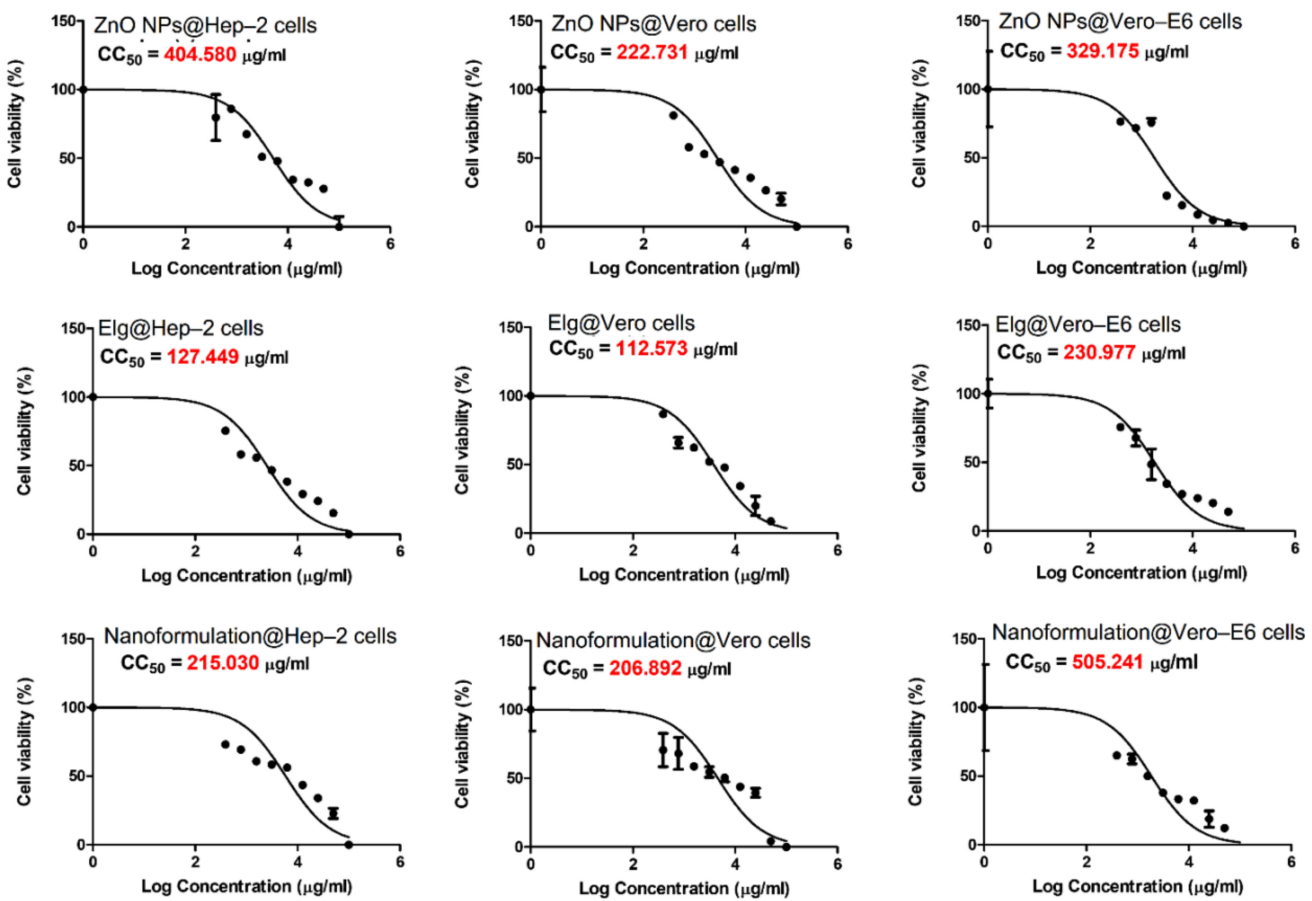
The cytotoxic effects (CC50) of tested materials against three cell lines for the investigated viruses: Hep–2 cells, Vero cells, and Vero–E6 cells. Each graph was obtained by nonlinear regression analysis. We plotted log concentrations against cell viability normalized response.

The CC50 of the tested materials against the investigated three cell lines are shown in Figure 8. The cytotoxicity against Hep–2 cells was detected in the order ELG > nanoformulation > ZnO NPs. In the case of Vero cells, ELG had higher inhibition than the nanoformulation and ZnO NPs, respectively. For Vero–E6 cells, the cytotoxicity was detected in the order ELG > ZnO NPs > nanoformulation. This observation confirms that ELG has greater cytotoxic effects alone. The CC50 determined for each material was used for the antiviral investigation.

### 3.9. Antiviral Evaluation

**Figure 9:**
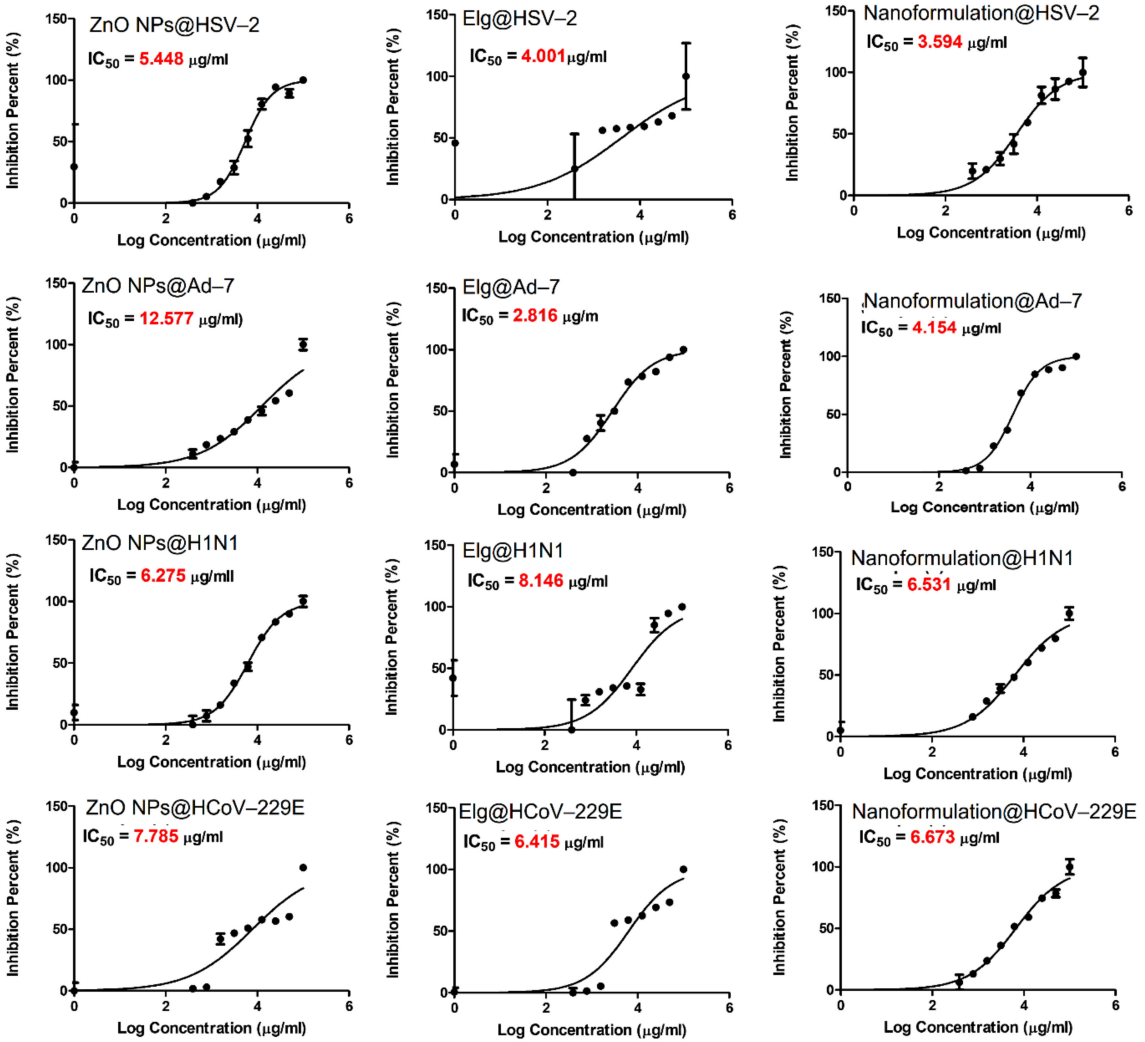
The antiviral effects (IC50) of tested materials against DNA and RNA viruses. DNA viruses were Ad-7 and HSV-2, and the RNA viruses were H1N1 and HCoV-229E. Each graph was obtained by nonlinear regression analysis. We plotted log concentrations against cell viability normalized response.

**Figure 10:**
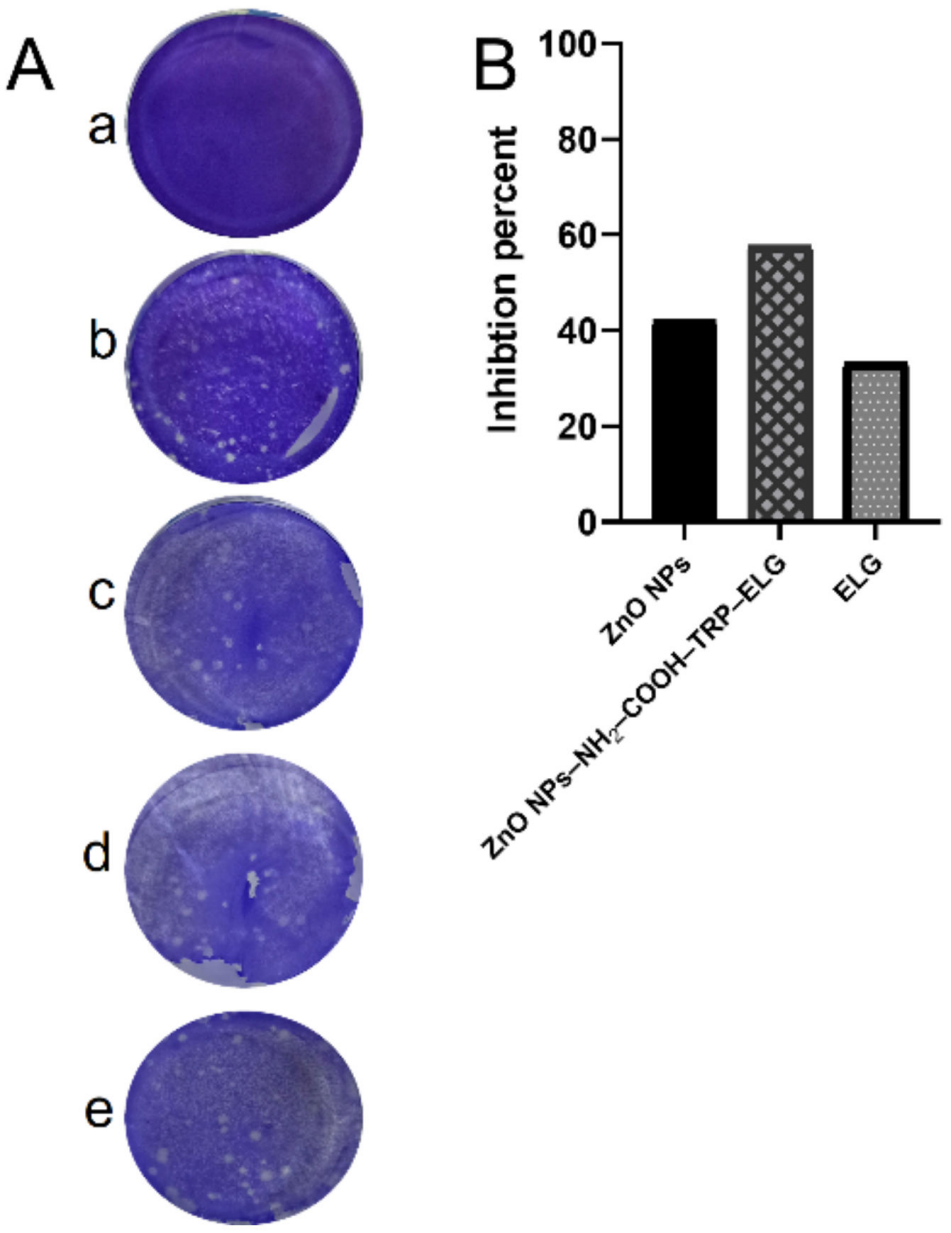
The virucidal mechanism and percent inhibition against coronavirus (HCoV-229E). (**A**) The cytotoxicity control (a), virus control (b), nanoformulation (c), ZnO NPs (d), and ELG (e). (**B**) The percent inhibition of coronavirus (CoV–229E) was calculated from the plaques formed through the virucidal mechanism.

Figure 9 shows the antiviral activity assessed by the CPE of each material against DNA viruses and RNA viruses. As a general trend, the IC50s varied in response to both treatment and virus. Interestingly, the findings indicate specific inactivity, depending on whether it was a DNA or RNA virus. In the case of DNA-based viruses, for HSV-2, the nanoformulation had higher virus inactivation (lowest IC50 of 3.59 µg/mL) than the ZnO NPs and ELG alone. For Ad-7, ELG recorded the lowest IC50 (2.82 µg/mL). In the case of RNA-based viruses, ZnO NPs inhibited viral infection by H1N1, with an IC50 of 6.27 µg/mL, compared to 6.53 µg/mL for the nanoformulation and 8.14 µg/mL for ELG. For HCoV-229E, ELG exhibited the lowest IC50 of 6.41 µg/mL, compared 6.67 µg/mL for nanoformulation and 7.78 µg/mL for ZnO NPs. To identify the most effective materials, we calculated a selectivity index (SI), the CC50/IC50 ratio (Table 2). The SI confirms the importance of specific inhibition of viral infections. The nanoformulation was a better treatment for the tested RNA viruses than the DNA viruses because the SI values were 77.3 and 75.7 for H1N1 and HCoV-229E, respectively, vs. 57.5 and 51.7 for HSV-2 and Ad-7, respectively. Therefore, the combination of ELG and ZnO NPs is important, as it enhanced the antiviral activity of the nanoformulation. We further tested the mechanism of action for the tested materials, especially on HCoV-229E, employing the virucidal mechanism (Figure 10). Interestingly, the nanoformulation inhibited plaques of HCoV-229E virus better than ZnO NPs and ELG alone (Figure 10A). These results not only confirm the data obtained by the CPE assays but also the efficiency of the nanoformulation. As indicated in Figure 10B, the nanoformulation significantly inhibited the formation of plaques of HCoV-229E, by ~60% (ZnO NPs ~42% and ELG ~33.5%). Thus, the nanoformulation can interact directly with HCoV-229E, resulting in a virucidal mechanism of action.

These findings are in line with our previous data [9], the proposed action for the virucidal mechanism can be related to surface chemistry of nanoformulation with carboxylic groups (from ELG) that have interacted with amine groups of the glycoproteins available on the virus surface. Based on the extensive research to screen several clinical drugs or explore new compounds against COVID-19, HCoV-229E is a good primary virus model and could be suitable for exploring specific anti-COVID-19 activity [50]. In this context, Parang et al. [50] evaluated remdesivir, which is an anti-HIV agent, and demonstrated that it inhibits HCoV-229E at an IC50 of 0.07 µM with an SI > 28 µM. Furthermore, Cheng et al. [51] studied the plant-derived saikosaponins and revealed potent anti-coronaviral activity of saikosaponin B2 against HCoV-229E at ~1.7 µg/L, with an SI > 200 µg/L through interference in the early viral replication stage, including in the absorption and penetration of the virus. It seems that the antiviral efficiency of either drugs or natural agents can vary; however, our findings are promising because of the low IC50 and high SI obtained against RNA viruses, especially HCoV-229E. Additionally, the use of ZnO NPs in the nanoformulation can also allow various routes of application for viral infections.

## 4. Conclusions

We developed an inorganic–organic hybrid nanoformulation, composed of zinc oxide NPs, functionalized with TRP (3D organic molecules) and loaded with ELG. The nanoformulation contained >33 wt.% ELG. The nanoformulation efficiently inhibited the RNA viruses H1N1 and HCoV-229E, as well as DNA viruses. Furthermore, the nanoformulation resulted in direct inactivation of HCoV-229E. The nanoformulation was significantly more efficient than the ZnO NPs or ELG when tested separately. The nanoformulation is a potentially safe and low-cost natural agent against coronavirus and H1N1, as well as other RNA-based viruses, and has potential as an alternative therapeutic strategy for COVID-19. However, further studies are needed to explore in vitro drug release (as well as in vivo studies), molecular targets, and other antiviral mechanisms towards the practical applications.

## Figures and Tables

**Table 1:** Physicochemical characteristics and drug loading properties of the prepared materials and nanoformulations.

Materials and Formulation	Weight Loss, wt.% ^a^	S_BET_ (m^2^/g) ^b^	Total Drug Content ^c^	Entrapment Efficiency, % ^d^
ZnO NPs	2.2	51.2		
ZnO NPs–NH_2_–COOH	3.3	21.6		
ZnO NPs–NH_2_–COOH–TRP	12.1	13.1		
ZnO NPs–NH_2_–COOH–TRP–ELG	45.5	11.7	33.3	83.2

^a^ Weight loss was calculated by the thermogravimetric analysis. ^b^ Specific surface area was measured using the BET technique. ^c,d^ Mean total drug loading and the entrapment efficiency were calculated from weight loss.

**Table 2:** The therapeutic effects calculated from the CC50 and IC50 of different materials.

Treatment/Virus	CC50, µg/mL	IC50, µg/mL	Therapeutic Index
ZnO NPs			
HSV-2	222.7	5.4	40.8
Ad-7	404.5	12.5	32.1
H1N1	329.1	6.2	52.4
HCoV-229E	329.1	7.7	42.3
ELG			
HSV-2	112.5	4.0	28.1
Ad-7	127.4	2.8	45.2
H1N1	230.9	8.1	28.3
HCoV-229E	230.9	6.4	36.0
ZnO NPs–NH_2_–COOH–TRP–ELG			
HSV-2	206.9	3.6	57.5
Ad-7	215.0	4.1	51.7
H1N1	505.2	6.5	77.3
HCoV-229E	505.2	6.6	75.7

## Data Availability

The data presented in this study are available upon request from the corresponding author.
